# Occupational Stress, Burnout, and Depression in Women in Healthcare During COVID-19 Pandemic: Rapid Scoping Review

**DOI:** 10.3389/fgwh.2020.596690

**Published:** 2020-11-26

**Authors:** Abi Sriharan, Savithiri Ratnapalan, Andrea C. Tricco, Doina Lupea, Ana Patricia Ayala, Hilary Pang, Dongjoo Daniel Lee

**Affiliations:** ^1^Institute of Health Policy, Management and Evaluation, University of Toronto, Toronto, ON, Canada; ^2^Dalla Lana School of Public Health, University of Toronto, Toronto, ON, Canada; ^3^Department of Pediatrics, University of Toronto, Toronto, ON, Canada; ^4^Hospital for Sick Children, Toronto, ON, Canada; ^5^Li Ka Shing Knowledge Institute, St. Michael's Hospital, Unity Health Toronto, Toronto, ON, Canada; ^6^Joanna Briggs Institute Centre of Excellence, Queen's University, Kingston, ON, Canada; ^7^Physician Health Program, Ontario Medical Association, Toronto, ON, Canada; ^8^Gerstein Science Information Centre, University of Toronto, Toronto, ON, Canada; ^9^Faculty of Medicine, University of Toronto, Toronto, ON, Canada

**Keywords:** women, health care, occupational stress, burnout, mental health, pandemic, COVID-19, health work force

## Abstract

**Objectives:** The overall objectives of this rapid scoping review are to (a) identify the common triggers of stress, burnout, and depression faced by women in health care during the COVID-19 pandemic, and (b) explore individual-, organizational-, and systems-level interventions that can support the well-being of women HCWs during a pandemic.

**Design:** This scoping review is registered on Open Science Framework (OSF) and was guided by the JBI guide to scoping reviews and reported using the Preferred Reporting Items for Systematic reviews and Meta-Analysis (PRISMA) extension to scoping reviews. A systematic search of literature databases (Medline, EMBASE, CINAHL, PsycInfo and ERIC) was conducted from inception until June 12, 2020. Two reviewers independently assessed full-text articles according to predefined criteria.

**Interventions:** We included review articles and primary studies that reported on stress, burnout, and depression in HCWs; that primarily focused on women; and that included the percentage or number of women included. All English language studies from any geographical setting where COVID-19 has affected the population were reviewed.

**Primary and secondary outcome measures:** Studies reporting on mental health outcomes (e.g., stress, burnout, and depression in HCWs), interventions to support mental health well-being were included.

**Results:** Of the 2,803 papers found, 28 were included. The triggers of stress, burnout and depression are grouped under individual-, organizational-, and systems-level factors. There is a limited amount of evidence on effective interventions that prevents anxiety, stress, burnout and depression during a pandemic.

**Conclusions:** Our preliminary findings show that women HCWs are at increased risk for stress, burnout, and depression during the COVID-19 pandemic. These negative outcomes are triggered by individual level factors such as lack of social support; family status; organizational factors such as access to personal protective equipment or high workload; and systems-level factors such as prevalence of COVID-19, rapidly changing public health guidelines, and a lack of recognition at work.

## Strengths and Limitations of this Study

- A rapid scoping review was conducted to identify stress, burnout and depression faced by women HCWs during COVID-19.
- To ensure the relevance of our review, representatives from the women HCWs were engaged in defining the review scope, developing review questions, approving the protocol and literature search strategies, and identifying key messages.
- It provides a descriptive synthesis of current evidence on interventions to prevent mental health for women HCWs.
- Most studies used cross-sectional surveys, making it difficult to determine the longitudinal impact.
- There was significant variability in the tools used to measure mental health.

## Introduction

COVID-19 pandemic-related measures, such as prolonged periods of social isolation, unexpected employment disruptions, school closures, financial distress, and changes to routine, are having an unprecedented negative impact on women's mental well-being (UN). Over 80% of the health workers in Canada are women (1). Women in health care already face systemic challenges related to workplace gender biases, discrimination, sexual harassment, and other inequities (2). Studies show that women physicians are more likely than male physicians to experience depression, burnout, and suicidal ideation (3, 4). Additionally, women perform three times more unpaid care work than men as parents and primary caregivers to family members (5).

The COVID-19 pandemic has led to increased psychological trauma and suicide among health care workers (HCWs) (6–10). A poll of HCWs conducted by the Public Health Agency of Canada in April 2020 showed that 47% of respondents expressed the need for psychological support due to COVID-19 related factors; 90% of the respondents were women (11). Similarly, a survey conducted by the British Medical Association in April, 2020 of HCWs showed that 44% of respondents indicated they were experiencing burnout, depression, anxiety, or other mental health conditions due to COVID-19-related factors (12). Unaddressed stress and burnout can lead to depression, suicidal ideation, and substance abuse (4, 13). A healthy workforce is the cornerstone of a well-functioning health care system. Yet, there is a systemic lack of evidence-informed services that provide timely, accessible, and high-quality care for HCWs during public health crises. This is especially relevant for health systems and professional societies who recognize the importance of preventing and mitigating stress, burnout, depression, and suicidal ideation in their workforce during pandemics. In addition, these interventions are essential for the well-being and retention of the health care workforce. This review attempts to answer the following questions: What are the common triggers of occupational stress, burnout, and depression faced by women in health care during the COVID-19 pandemic? What individual-, organizational-, and systems-level interventions can support the well-being of women HCWs during a pandemic?

### Overall Objectives

The overall objectives of this review are to (a) identify the common triggers of occupational stress, burnout, and depression faced by women in health care during the COVID-19 pandemic, and (b) explore individual-, organizational-, and systems-level interventions that can support the well-being of women HCWs during a pandemic.

## Methods

### Commissioning Agency

The Canadian Institute for Health Research issued a special call to address COVID-19 in Mental Health & Substance Use issues. Given there has not been any previous research in this topic area, and the need to provide decision-makers with timely results, a rapid scoping review was conducted in accordance with the WHO Rapid Review Guide and the JBI 2020 guide to scoping reviews (14, 15) and reported using Preferred Reporting Items for Systematic Reviews and Meta-Analyses (PRISMA) for scoping reviews. Scoping reviews help map the key concepts and underpin a field of research and clarify working definitions (16, 17).

### Protocol

This review is registered with the Open Science Framework (https://osf.io/y8fdh/?view_only=1d943ec3ddbd4f5c8f6a9290eca2ece7).

### Eligibility Criteria

The following PICOS (population, intervention, comparator, outcome, Study Design) eligibility criteria were developed a priori:

#### Population

Women HCWs. We define HCWs as “all people engaged in actions whose primary intent is to enhance health,” (18). This encompasses a broad array of health workers, including doctors, nurses, pharmacists, midwives, paramedics, physical therapists, technicians, personnel support workers, and community health workers. We included studies that primarily focused on women.

#### Interventions

Our inclusion criteria were all studies (primary and review articles) that reported on the causes of stress, burnout, and depression in HCWs and/or reported programs to mitigate stress, burnout, and Depression in HCWs.

#### Comparators

Not applicable for the purpose of this scoping review.

#### Outcomes

We looked at following outcomes: stress, burnout, and depression. We define stress as the degree to which one feels overwhelmed and unable to cope as a result of unmanageable pressures (19). We define burnout as the experience of emotional exhaustion, depersonalization, or cynicism, along with feelings of diminished personal efficacy or accomplishment in the context of the work environment (20). We characterize depression according to a series of symptoms, including low mood, changes in appetite and sleep, difficulty concentrating, loss of interest/pleasure and thoughts of suicide that persist for at least 2 weeks (21).

#### Study Design

We included review articles and primary studies where data were collected and analyzed using quantitative, qualitative, and mixed methods (14). We excluded editorials and opinion pieces unless the authors shared their personal experiences.

### Search Methods and Information Sources

We conducted comprehensive search strategies in the following electronic databases: Medline (via OVID), Embase (via Ovid), CINAHL (via EBSCOHost), PsycINFO (via Ovid), and ERIC (via ProQUEST). Search strategies were developed by an academic health sciences librarian (APA), with input from the research team. The search was original built in MEDLINE Ovid, and peer reviewed using the Peer Review of Electronic Search Strategies (PRESS) tool (22), before being translated into other databases using their command language if applicable. The Coronavirus (Covid-19) 2019-nCov expert search from Ovid MEDLINE was used and translated to other databases. Searches were limited to articles published until June 12th, 2020, and by English language. The final search results were exported into Covidence, a review management software, where duplicates were identified and removed.

### Screening Process

To minimize selection bias, we piloted 20 articles against *a priori* inclusion and exclusion criteria. Each article title was reviewed by two independent screeners against using Covidence. A third reviewer reviewed conflicts and resolved disagreements through discussion. Two reviewers also independently screened the full text of potentially eligible articles to check whether the articles fulfilled the inclusion criteria.

### Data Charting

We used a predefined data extraction form to extract data from the papers included in the review. To ensure the integrity of the assessment, we piloted the data extraction form on three studies. We extracted the following information from the studies: the first author, year of publication, HCWs enrolled in the study, geographic location, study methods, and intervention information that could help answer our objectives. Scoping reviews are conducted to provide an overview of the existing evidence regardless of methodological quality or risk of bias. As a standard, included sources of evidence are not critically appraised for scoping reviews (14, 15). In accordance with this we did not appraise quality or risk of bias of the included articles. Ethical approval was not required for this review.

### Data Synthesis

Due to heterogeneity regarding outcome measurement and statistical analysis, data was descriptively synthesized.

### Patient Involvement

No patients were involved in setting the research question or the outcome measures, nor were they involved in the design and implementation of the study.

## Results

### Search Results

**Figure 1:**
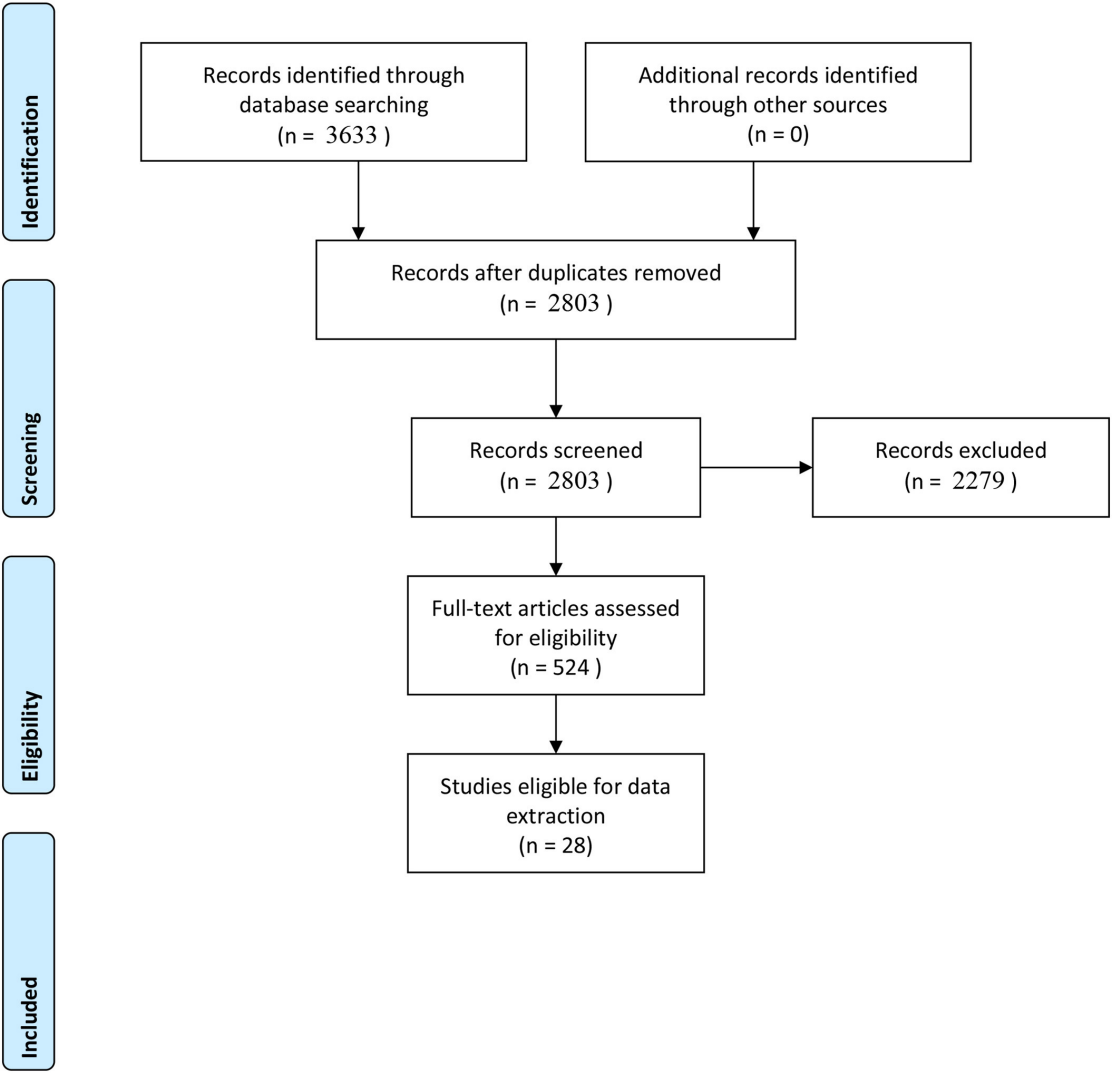
PRISMA 2009 flow diagram.

The search resulted in a total of 3,633 records. After 830 duplicates were removed, 2,803 records remained to be screened. We excluded 2,279 records based on title and abstract screening. We assessed 524 full-text articles. Most of these articles are opinion pieces and commentaries. Twenty eight published studies met our inclusion criteria and were included in this review. Figure 1 provides a summary of the PRISM flow diagram.

### Characteristics of Studies

Our search identified 28 eligible studies; 26 of these studies focused on the prevalence of mental health issues in health care professionals (Table 1). Two studies were case studies (Table 1). Sixteen of the primary studies were conducted in China, whereas others were conducted in Saudi Arabia, Italy, Singapore, India, and Colombia. These studies primarily focused on doctors, nurses, and generalized groups of allied health professionals. One study focused on dentists, whereas another focused-on pharmacists. The study samples included both male and women health professionals. Only one study focused exclusively on women in health care (23). Anxiety, depression, stress/distress symptoms, post-traumatic stress disorder, and insomnia were commonly assessed mental health issues in these studies.

**Table 1:** Summary of primary studies.

**Author**	**Study sample**	**Study type**	**Female**	**Doctors**	**Nurses**	**Other HCWs**	**Country**
1. Al Sulais	529	Cross-sectional Survey (CSS)	Breakdown N.A	X	–	–	Saudi Arabia
2. Almagharabi	1,036	CSS					Saudi Arabia
3. Cai	534	CSS	367	X			China
4. Chew	906	CSS	Breakdown Not Available (N.A)	X	(N.A.)	(N.A.)	China
5. Chowdhury, S,M		Qualitative					
6. DeStefani	1,500		836	–	–	Dentists	Italy
7. Elbay	442	CSS	251	X	–	–	Turkey
8. Felice	388	CSS	235	X	–	X	Italy
9. Huang	600	CSS	305	X	X	X	China
10. Kang	994	CSS	850	X	X	–	China
11. Karasneh	486	CSS	382	–	–	X	Jordan
12. Khanna	2,355	CSS	1,332	X	–	–	India
13. Lai	1,257	CSS	964	X	X	–	China
14. Li	5,317	CSS	5,317	X	X	X	China
15. Liu	512	CSS	433	N.A.	N.A.	N.A.	China
16. Marton		Qualitative					
17. Pedrozo-Papa	179	CSS	Breakdown N.A	X	X	X	Columbia
18. Romero	1,671	CSS	Breakdown N.A	X	–	–	Spain
19. Song	14,825	CSS	9,536	X	X	–	China
20. Sun	442	CSS	368	X	X	X	China
21. Uzun	103	CSS	91	X	X	X	Turkey
22. Wu	190	CSS	157	–	X	X	China
23. Xiao	958	CSS	644	X	X	X	China
24. Yin	371	CSS	228	X	X	X	China
25. Yuan	939	CSS	582	X	–	X	China
26. Zhang	304	CSS	178	N.A.	N.A.	N.A.	China
27. Zhang	927	CSS	678	X	X	X	China
28. Zhu	165	CSS	137	X	X	–	China

A variety of assessment tools were used to measure mental health in these studies. Common tools used to measure psychosocial well-being included DASS-21, Impact of Event Scale Revised Questionnaire (IES-R), Connor-Davidson Resilience Scale, Chinese Perceived Stress Scale, Patient Health Questionnaire-9 (PHQ-9), Generalized Anxiety Disorder (GAD-7) Scale, Questionnaire Star, Psychological Symptom Screening Test (SCL-90-R), Beck Anxiety Inventory and Short Psychiatric Rating Scale, Maslach Burnout Inventory-Medical Personnel, Perceived Stress Scale and Hospital Anxiety/Depression Scale, Post-traumatic Stress Disorder Checklist for DSM-5 and the Pittsburgh Sleep Quality Index, Stress Response Questionnaire, Zung Self-Rating Anxiety Scale SF-12, K6, Insomnia Severity Index, Self-Rating Depression Scale, and Simplified Coping Style Questionnaire.

### Common Triggers of Stress, Burnout, and Depression Faced by Women in Health Care During the Coronavirus Pandemic

Common triggers of mental health issues were fears of getting infected with COVID-19 and putting family members at risk (24–26), as well as concerns about professional growth, difficulty meeting living expenses (27), and having family members with suspected and confirmed COVID-19 (23). Individual-, organizational-, and systems-level factors are reported as common triggers of stress, burnout, and depression in women HCWs.

#### Individual-Level Factors

Women HCWs are more likely than men HCWs to experience psychological stress and burnout (24, 28–36). More specifically, young women HCWs and mid-career women HCWs were more likely to experience emotional and mental health issues due to COVID-19 (23, 29, 37). Similarly, less working experience and self-perception about lack of competency to care for COVID-19 patients was associated with increased prevalence of stress and burnout (29, 37). Women who are single or lacking social support are more at risk of developing symptoms of anxiety, stress and burnout (23, 29, 37–39). Women HCWs with medical or psychiatric comorbidities (23, 39) or increased alcohol use are at higher risk of mental health issues (37). Surprisingly, women HCWs who have more than two children experience higher prevalence of psychosocial well-being (29).

#### Organizational-Level Factors

Long working hours and increased workload (29, 37, 40); increased number of COVID-19 patients under their care (29, 41); lack of access to personal protective equipment (25, 26, 28, 30, 40, 42–44); lack of infection control guidelines and protocols (26, 29, 42, 45); lack of support and recognition by their peers, supervisors, and hospital leadership (26, 29); and work location (30, 43) are reported as common triggers of mental health issues related to the work environment.

#### Systems-Level Factors

Increased incidence of COVID-19 cases in the local area (26), changes in public health measures and guidelines (46), information shared in the media (47), and lack of recognition by the government officials and policy makers of HCWs' work conditions (26) are reported to increase stress and mental health issues among HCWs.

### Interventions That Can Support the Well-Being of Women HCWs During a Pandemic

Very few studies have discussed potential interventions to support women in health care with COVID-19 related stress, anxiety, and mental health. Women with increased workloads preferred to use psychological support (40). Regular exercise is considered a protective factor for depression and anxiety (23). Time is considered a modifiable factor that improves anxiety level (32). Mental health services such as online resources, psychological assistance hotlines, and group activities for stress reduction are poorly utilized by HCWs (38). Online-push messages of mental health self-help and self-help books are mostly preferred by women HCWs (38). Measures to support HCWs financially (25), provision of rest areas for sleep and recovery (Yin, 29), care for basic physical needs such as food (38), training programs to improve resiliency (33), information on protective measures (38), and access to leisure activities (38) and counselors (38, 41) are considered potential strategies to support HCWs during a pandemic. However, these studies did not measure the impact of these interventions.

## Discussion

This review shows that individual characteristics such as sex (women), age (younger women), marital status (single women and women with young children), and career stage (less experience) have been contributing factors to occupational stress, burnout, and depression during COVID-19. The current literature lacks data on how socioeconomic, cultural, and ethnoracial differences influence occupational stress, burnout, and depression in women HCWs.

At the organizational level, lack of training, poor infection control guidelines, work conditions that include changing policies, higher workload, and inadequate access to personal protective equipment are contributing to occupational stress, burnout, and depression in women during COVID-19. The long-term effects of burnout during COVID-19 are unknown. General studies on burnout in HCWs has shown an association between burnout and poor career satisfaction, high absenteeism, career transitions, early retirements, and familial and marital stressors (48, 49).

This review shows there is relatively little empirical research into possible interventions to help support women HCWs during a pandemic. Interventions to reduce occupational stress and burnout among HCWs have primarily focused on providing mental health services such as online resources and psychological assistance hotlines, the effects of which have been mixed. This is consistent with findings from HCW burnout studies unrelated to COVID-19. There is a lack of understanding about the effects of organizational interventions such as workload policies and procedures; organizational support systems, such as employee assistance programs; coaching and resiliency and mindfulness training programs, such as reducing working hours; caseloads; and on-call procedures.

Virtually all empirical studies included in this review are epidemiological studies of occupational stress, burnout, and depression. However, there was a significant variability in the tools used to measure stress, burnout, and depression. Further, the current literature has emerged from limited geographical regions. It is unclear how variations in health care and organizational and cultural contexts will shape the outcomes of similar studies carried out across a broader geographic area. During the search and screening, we specifically focused on including articles focused on female health workers or articles that included both genders. We noticed, often the gender-based analysis was clearly articulated in several publications.

This review covers limited empirical and review studies published on the topic of stress, burnout and health care workers from the start of COVID-19 until June 2020. As a scoping review, we were able to map the emerging concepts that underpin occupation stress and wellness for health care women during the COVID-19 pandemic. We expect to see an increased number of publications concerning COVID-19's impact on health professionals will emerge in the next 6 months. We have registered a rapid review protocol in the International Prospective Register of Systematic Reviews (PROSPERO CRD42020189750) to systematically examine the emerging evidence on occupational stress, burnout, and depression in HCWs during the COVID-19 pandemic.

## Conclusions

Women HCWs are at increased risk for occupational stress, burnout, and depression during the COVID-19 pandemic because of a combination of personal and organizational factors. However, there is a significant gap in the evidence base as to what interventions can help address these issues. We recommend that health-system decision-makers, hospitals, and professional organizations support research that measures the long-term impact of COVID-19 on women in health care and outcome studies that measure the impact of various mental health interventions and resources supporting women in health care. Given the complex nature of these interventions, we urge future researchers to provide the contexts in which the interventions were implemented and the mechanisms that shape successful interventions.

## Data Availability Statement

The original contributions presented in the study are included in the article/Supplementary Materials, further inquiries can be directed to the corresponding author/s.

## Author Contributions

AS, SR, AT, and DL conceptualized and designed the review. AS, AA, HP, and DDL reviewed titles, abstracts, and full-text papers for eligibility. AS, HP, and DDL were responsible for extracting data and all data extraction was verified by AS. AS prepared the initial draft manuscript. SR, AT, and DL reviewed and edited the manuscript. All authors contributed to the article and approved the submitted version.

## Conflict of Interest

The authors declare that the research was conducted in the absence of any commercial or financial relationships that could be construed as a potential conflict of interest.
