# Supplementary material for: Occupational Stress, Burnout, and Depression in Women in Healthcare During COVID-19 Pandemic: Rapid Scoping Review
Source: Front Glob Womens Health. 2020 Nov 26;1:596690. doi: 10.3389/fgwh.2020.596690 (PMC8594027; doi:10.3389/fgwh.2020.596690)
Supplement: Supplementary file 1 [file Table_1.DOCX]

Appendix A

Database(s): **Ovid MEDLINE: Epub Ahead of Print, In-Process & Other Non-Indexed Citations, Ovid MEDLINE® Daily and Ovid MEDLINE®**1946-Present
Search Strategy:

| **#** | **Searches** |
| --- | --- |
| 1 | exp Coronavirus/ |
| 2 | exp Coronavirus Infections/ |
| 3 | (coronavirus* or corona virus* or OC43 or NL63 or 229E or HKU1 or HCoV* or ncov* or covid* or sars-cov* or sarscov* or Sars-coronavirus* or Severe Acute Respiratory Syndrome Coronavirus*).mp. |
| 4 | (or/1-3) and ((20191* or 202*).dp. or 20190101:20301231.(ep).) [this set is the sensitive/broad part of the search] |
| 5 | 4 not (SARS or SARS-CoV or MERS or MERS-CoV or Middle East respiratory syndrome or camel* or dromedar* or equine or coronary or coronal or covidence* or covidien or influenza virus or HIV or bovine or calves or TGEV or feline or porcine or BCoV or PED or PEDV or PDCoV or FIPV or FCoV or SADS-CoV or canine or CCov or zoonotic or avian influenza or H1N1 or H5N1 or H5N6 or IBV or murine corona*).mp. [line 5 removes noise in the search results] |
| 6 | ((pneumonia or covid* or coronavirus* or corona virus* or ncov* or 2019-ncov or sars*).mp. or exp pneumonia/) and Wuhan.mp. |
| 7 | (2019-ncov or ncov19 or ncov-19 or 2019-novel CoV or sars-cov2 or sars-cov-2 or sarscov2 or sarscov-2 or Sars-coronavirus2 or Sars-coronavirus-2 or SARS-like coronavirus* or coronavirus-19 or covid19 or covid-19 or covid 2019 or ((novel or new or nouveau) adj2 (CoV on nCoV or covid or coronavirus* or corona virus or Pandemi*2)) or ((covid or covid19 or covid-19) and pandemic*2) or (coronavirus* and pneumonia)).mp. |
| 8 | COVID-19.rx,px,ox. or severe acute respiratory syndrome coronavirus 2.os. |
| 9 | ("32240632" or "32236488" or "32268021" or "32267941" or "32169616" or "32267649" or "32267499" or "32267344" or "32248853" or "32246156" or "32243118" or "32240583" or "32237674" or "32234725" or "32173381" or "32227595" or "32185863" or "32221979" or "32213260" or "32205350" or "32202721" or "32197097" or "32196032" or "32188729" or "32176889" or "32088947" or "32277065" or "32273472" or "32273444" or "32145185" or "31917786" or "32267384" or "32265186" or "32253187" or "32265567" or "32231286" or "32105468" or "32179788" or "32152361" or "32152148" or "32140676" or "32053580" or "32029604" or "32127714" or "32047315" or "32020111" or "32267950" or "32249952" or "32172715").ui. [Articles not captured by this search when created in April 2020, pending further indexing by NLM] |
| 10 | or/6-9 [Lines 6 to 9 are specific to Covid-19] |
| 11 | 5 or 10 |
| 12 | 11 and 20191201:20301231.(dt). [OVID Medline expert June 2] |
| 13 | ((wuhan or beijing or shanghai or korea or spain or portugal or italy or usa or uk or brazil or france or mexico) adj2 corona*).mp. |
| 14 | ((wuhan or beijing or shanghai or korea or spain or portugal or italy or USA or UK or brazil or france or mexico) adj2 pneumoni*).mp. |
| 15 | (wuhan or beijing or shanghai or korea or italy or spain or usa or uk or brazil or france or mexico).mp. and (exp coronavirus/ or exp pneumonia/) |
| 16 | (exp china/ or exp korea/ or exp Europe/ or north america/ or canada/ or mexico/ or united states/) and (exp coronavirus/ or exp pneumonia/) |
| 17 | ("corona viru*" or "nCov" or "2019 ncov" or "Novel Coronavirus" or "covid-19" or "SARS-COV-2" or "Severe Acute Respiratory Syndrome Coronavirus 2" or "coronavirus disease 2019" or "corona virus disease 2019").tw,kf. |
| 18 | ("new coronaviru*" or "2019 ncov" or "nCov 2019" or "SARS Coronavirus 2" or "novel coronaviru*").mp. |
| 19 | "Severe Acute Respiratory Syndrome Coronavirus 2".mp. |
| 20 | ((new or novel or emerging or "2020") adj2 (coronoviru* or "corona viru*")).tw,kf. |
| 21 | ((pandemic or epidemic or "global spread") adj3 (coronavir* or corona or covid or "2020")).mp. |
| 22 | ("2020" adj2 (pandemic or epidemic or "global disease spread" or "virus spread")).mp. |
| 23 | ("Novel Corona virus" or "corona virus disease 2019" or "nCov 2019" or "SARS Coronavirus 2" or "COVID-2019" or nCoV or "2019-nCoV" or covid19 or " covid-19" or "SARS-CoV-2").tw,kf. |
| 24 | coronavir*.mp. |
| 25 | or/12-24 [COVID textwords + MesH] |
| 26 | burnout, psychological/ or burnout, professional/ or occupational stress/ or compassion fatigue/ |
| 27 | ((stress* or anxiety or anxious or burnout or burn-out or depress* or overwhelm* or "psychological workload" or fatigue* or exhaust*) adj4 (occupation* or work or profession* or job)).tw,kf. |
| 28 | Stress, Psychological/ |
| 29 | (behavio?r* or stress* or adaptation or psycholog* or psychological or emotion* or depress* or attitude* or sleep or burnout or fatigue* or compassion or resilience or resilient* or anxiety or anxious* or exhaust* or mental).tw,kf. |
| 30 | exp Mental Disorders/ or sleep/ or sleep deprivation/ or sleep hygiene/ or stress, psychological/ or burnout, psychological/ or burnout, professional/ or historical trauma/ or occupational stress/ or compassion fatigue/ or resilience, psychological/ or mental health/ or behavioral symptoms/ or depression/ or mental fatigue/ or alert fatigue, health personnel/ |
| 31 | px.fs. |
| 32 | physicians/ or allergists/ or anesthesiologists/ or cardiologists/ or dermatologists/ or endocrinologists/ or foreign medical graduates/ or gastroenterologists/ or general practitioners/ or geriatricians/ or hospitalists/ or nephrologists/ or neurologists/ or occupational health physicians/ or exp oncologists/ or ophthalmologists/ or osteopathic physicians/ or otolaryngologists/ or pathologists/ or exp pediatricians/ or physiatrists/ or physicians, family/ or physicians, primary care/ or physicians, women/ or pulmonologists/ or exp radiologists/ or rheumatologists/ or exp surgeons/ or urologists/ or exp medicine/ or exp Medical Staff, Hospital/ |
| 33 | health personnel/ or allied health personnel/ or animal technicians/ or community health workers/ or dental auxiliaries/ or dental assistants/ or dental hygienists/ or dental technicians/ or denturists/ or emergency medical technicians/ or home health aides/ or licensed practical nurses/ or medical record administrators/ or medical secretaries/ or medical receptionists/ or nursing assistants/ or psychiatric aides/ or operating room technicians/ or pharmacy technicians/ or physical therapist assistants/ or physician assistants/ or ophthalmic assistants/ or pediatric assistants/ or anatomists/ or anesthetists/ or anesthesiologists/ or nurse anesthetists/ or audiologists/ or caregivers/ or case managers/ or "coroners and medical examiners"/ or dental staff/ or dental staff, hospital/ or dentists/ or dentists, women/ or endodontists/ or "oral and maxillofacial surgeons"/ or orthodontists/ or doulas/ or emergency medical dispatcher/ or epidemiologists/ or faculty, dental/ or faculty, medical/ or faculty, nursing/ or health educators/ or health facility administrators/ or hospital administrators/ or chief executive officers, hospital/ or infection control practitioners/ or medical chaperones/ or medical laboratory personnel/ or medical staff/ or medical staff, hospital/ or hospitalists/ or nurses/ or nurse administrators/ or nurse practitioners/ or family nurse practitioners/ or pediatric nurse practitioners/ or nurse specialists/ or nurse clinicians/ or nurse midwives/ or nurses, pediatric/ or nurses, neonatal/ or nurses, community health/ or nurses, international/ or nurses, male/ or nurses, public health/ or nursing staff/ or nursing staff, hospital/ or nutritionists/ or occupational therapists/ or optometrists/ or personnel, hospital/ or hospital volunteers/ or pharmacists/ or physical therapists/ or physician executives/ |
| 34 | (physician* or counsellor* or psychologist* or doctor* or clinician* or nurs* or hospitalist* or radiologists* or nephrologist* or therapist* or "medical staff" or cardiologist* or oncologist* or nutritionist* or pharmacist* or ophthalmologist* or otolaryngologist* or surgeon* or urologist* or epidemiologist* or pathologist* or physiatrist* or geriatrician* or gerontologist* or dentist* or doula* or anesthesiologist* or dermatologist*).tw,kf. |
| 35 | ((hospital* or clinical or medical or health*) adj3 (staff or personnel or employee* or worker* or professional*)).tw,kf. |
| 36 | or/12,25 [Covid] |
| 37 | exp health personnel/ or exp laboratory personnel/ |
| 38 | 32 or 33 or 34 or 35 or 37 [Health personnel] |
| 39 | or/26-31 [Burnout, stress, depression, anxiety] |
| 40 | 36 and 38 and 39 |
| 41 | limit 40 to yr="2003 -Current" |
| 42 | limit 41 to english language |

Database(s): **Embase Classic+Embase**1947 to 2020 June 19
Search Strategy:

| **#** | **Searches** |
| --- | --- |
| 1 | coronavirinae/ or coronaviridae/ |
| 2 | (coronavirus* or corona virus* or OC43 or NL63 or 229E or HKU1 or HCoV* or ncov* or covid* or sars-cov* or sarscov* or Sars-coronavirus* or Severe Acute Respiratory Syndrome Coronavirus*).mp. |
| 3 | (or/1-2) and (20191* or 202*).dp. [this set is the sensitive/broad part of the search] |
| 4 | 3 not (SARS or SARS-CoV or MERS or MERS-CoV or Middle East respiratory syndrome or camel* or dromedar* or equine or coronary or coronal or covidence* or covidien or influenza virus or HIV or bovine or calves or TGEV or feline or porcine or BCoV or PED or PEDV or PDCoV or FIPV or FCoV or SADS-CoV or canine or CCov or zoonotic or avian influenza or H1N1 or H5N1 or H5N6 or IBV or murine corona*).mp. [line 5 removes noise in the search results] |
| 5 | ((pneumonia or covid* or coronavirus* or corona virus* or ncov* or 2019-ncov or sars*).mp. or exp pneumonia/) and Wuhan.mp. |
| 6 | (2019-ncov or ncov19 or ncov-19 or 2019-novel CoV or sars-cov2 or sars-cov-2 or sarscov2 or sarscov-2 or Sars-coronavirus2 or Sars-coronavirus-2 or SARS-like coronavirus* or coronavirus-19 or covid19 or covid-19 or covid 2019 or ((novel or new or nouveau) adj2 (CoV on nCoV or covid or coronavirus* or corona virus or Pandemi*2)) or ((covid or covid19 or covid-19) and pandemic*2) or (coronavirus* and pneumonia)).mp. |
| 7 | ("32240632" or "32236488" or "32268021" or "32267941" or "32169616" or "32267649" or "32267499" or "32267344" or "32248853" or "32246156" or "32243118" or "32240583" or "32237674" or "32234725" or "32173381" or "32227595" or "32185863" or "32221979" or "32213260" or "32205350" or "32202721" or "32197097" or "32196032" or "32188729" or "32176889" or "32088947" or "32277065" or "32273472" or "32273444" or "32145185" or "31917786" or "32267384" or "32265186" or "32253187" or "32265567" or "32231286" or "32105468" or "32179788" or "32152361" or "32152148" or "32140676" or "32053580" or "32029604" or "32127714" or "32047315" or "32020111" or "32267950" or "32249952" or "32172715").ui. [Articles not captured by this search when created in April 2020, pending further indexing by NLM] |
| 8 | or/5-7 [Lines 5-7 specific to Covid-19] |
| 9 | 4 or 8 |
| 10 | ((wuhan or beijing or shanghai or korea or spain or portugal or italy or usa or uk or brazil or france or mexico) adj2 corona*).mp. |
| 11 | ((wuhan or beijing or shanghai or korea or spain or portugal or italy or USA or UK or brazil or france or mexico) adj2 pneumoni*).mp. |
| 12 | (wuhan or beijing or shanghai or korea or italy or spain or usa or uk or brazil or france or mexico).mp. and (exp coronavirinae/ or exp pneumonia/) |
| 13 | exp China/ |
| 14 | exp Korea/ |
| 15 | exp Europe/ |
| 16 | north america/ or exp canada/ or exp mexico/ or exp united states/ |
| 17 | 13 or 14 or 15 or 16 |
| 18 | exp coronavirinae/ or exp pneumonia/ |
| 19 | 17 and 18 |
| 20 | ("corona viru*" or "nCov" or "2019 ncov" or "Novel Coronavirus" or "covid-19" or "SARS-COV-2" or "Severe Acute Respiratory Syndrome Coronavirus 2" or "coronavirus disease 2019" or "corona virus disease 2019").tw,kw. |
| 21 | ("new coronaviru*" or "2019 ncov" or "nCov 2019" or "SARS Coronavirus 2" or "novel coronaviru*").mp. |
| 22 | "Severe Acute Respiratory Syndrome Coronavirus 2".mp. |
| 23 | ((new or novel or emerging or "2020") adj2 (coronoviru* or "corona viru*")).tw,kw. |
| 24 | ((pandemic or epidemic or "global spread") adj3 (coronavir* or corona or covid or "2020")).mp. |
| 25 | ("2020" adj2 (pandemic or epidemic or "global disease spread" or "virus spread")).mp. |
| 26 | ("Novel Corona virus" or "corona virus disease 2019" or "nCov 2019" or "SARS Coronavirus 2" or "COVID-2019" or nCoV or "2019-nCoV" or covid19 or " covid-19" or "SARS-CoV-2").tw,kw. |
| 27 | coronavir*.mp. |
| 28 | 9 or 10 or 11 or 12 or 19 or 20 or 21 or 22 or 23 or 24 or 25 or 26 or 27 [Covid] |
| 29 | ((stress* or anxiety or anxious or burnout or burn-out or depress* or overwhelm* or "psychological workload" or fatigue* or exhaust*) adj4 (occupation* or work or profession* or job)).tw,kw. |
| 30 | (behavio?r* or stress* or adaptation or psycholog* or psychological or emotion* or depress* or attitude* or sleep or burnout or fatigue* or compassion or resilience or resilient* or anxiety or anxious* or exhaust* or mental).tw,kw. |
| 31 | exp burnout/ |
| 32 | exp stress/ |
| 33 | exp depression/ |
| 34 | exp mental disease/ |
| 35 | sleep hygiene/ |
| 36 | sleep deprivation/ |
| 37 | psychological resilience/ |
| 38 | compassion fatigue/ |
| 39 | exp health care personnel/ |
| 40 | (physician* or counsellor* or psychologist* or doctor* or clinician* or nurs* or hospitalist* or radiologists* or nephrologist* or therapist* or "medical staff" or cardiologist* or oncologist* or nutritionist* or pharmacist* or ophthalmologist* or otolaryngologist* or surgeon* or urologist* or epidemiologist* or pathologist* or physiatrist* or geriatrician* or gerontologist* or dentist* or doula* or anesthesiologist* or dermatologist*).tw,kw. |
| 41 | ((hospital* or clinical or medical or health*) adj3 (staff or personnel or employee* or worker* or professional*)).tw,kw. |
| 42 | or/29-38 [Mental health] |
| 43 | or/39-41 [Health personnel] |
| 44 | 28 and 42 and 43 |
| 45 | limit 44 to (english language and yr="2003 -Current") |

Database(s): **APA PsycInfo**1806 to June Week 3 2020
Search Strategy:

| **#** | **Searches** |
| --- | --- |
| 1 | (coronavirus* or corona virus* or OC43 or NL63 or 229E or HKU1 or HCoV* or ncov* or covid* or sars-cov* or sarscov* or Sars-coronavirus* or Severe Acute Respiratory Syndrome Coronavirus*).mp. |
| 2 | (pneumonia or covid* or coronavirus* or corona virus* or ncov* or 2019-ncov or sars*).mp. |
| 3 | pneumonia/ |
| 4 | wuhan.mp. |
| 5 | 2 or 3 |
| 6 | 4 and 5 |
| 7 | (2019-ncov or ncov19 or ncov-19 or 2019-novel CoV or sars-cov2 or sars-cov-2 or sarscov2 or sarscov-2 or Sars-coronavirus2 or Sars-coronavirus-2 or SARS-like coronavirus* or coronavirus-19 or covid19 or covid-19 or covid 2019 or ((novel or new or nouveau) adj2 (CoV on nCoV or covid or coronavirus* or corona virus or Pandemi*2)) or ((covid or covid19 or covid-19) and pandemic*2) or (coronavirus* and pneumonia)).mp. |
| 8 | ((wuhan or beijing or shanghai or korea or spain or portugal or italy or usa or uk or brazil or france or mexico) adj2 corona*).mp. |
| 9 | ((wuhan or beijing or shanghai or korea or spain or portugal or italy or USA or UK or brazil or france or mexico) adj2 pneumoni*).mp. |
| 10 | (wuhan or beijing or shanghai or korea or italy or spain or usa or uk or brazil or france or mexico).mp. and pneumonia/ |
| 11 | ("corona viru*" or "nCov" or "2019 ncov" or "Novel Coronavirus" or "covid-19" or "SARS-COV-2" or "Severe Acute Respiratory Syndrome Coronavirus 2" or "coronavirus disease 2019" or "corona virus disease 2019").mp. |
| 12 | ("new coronaviru*" or "2019 ncov" or "nCov 2019" or "SARS Coronavirus 2" or "novel coronaviru*").mp. |
| 13 | "Severe Acute Respiratory Syndrome Coronavirus 2".mp. |
| 14 | ((new or novel or emerging or "2020") adj2 (coronoviru* or "corona viru*")).mp. |
| 15 | ((pandemic or epidemic or "global spread") adj3 (coronavir* or corona or covid or "2020")).mp. |
| 16 | ("2020" adj2 (pandemic or epidemic or "global disease spread" or "virus spread")).mp. |
| 17 | ("Novel Corona virus" or "corona virus disease 2019" or "nCov 2019" or "SARS Coronavirus 2" or "COVID-2019" or nCoV or "2019-nCoV" or covid19 or " covid-19" or "SARS-CoV-2").mp. |
| 18 | coronavir*.mp. |
| 19 | 1 or 6 or 7 or 8 or 9 or 10 or 11 or 12 or 13 or 14 or 15 or 16 or 17 or 18 [Covid] |
| 20 | exp occupational stress/ or occupational health psychology/ or occupational neurosis/ or "quality of work life"/ or work related illnesses/ or work-life balance/ |
| 21 | "depression (emotion)"/ or exp major depression/ or sadness/ or sleep deprivation/ |
| 22 | exp mental disorders/ |
| 23 | "resilience (psychological)"/ |
| 24 | (behavio?r* or stress* or adaptation or psycholog* or psychological or emotion* or depress* or attitude* or sleep or burnout or fatigue* or compassion or resilience or resilient* or anxiety or anxious* or exhaust* or mental).tw,id. |
| 25 | ((stress* or anxiety or anxious or burnout or burn-out or depress* or overwhelm* or "psychological workload" or fatigue* or exhaust*) adj4 (occupation* or work or profession* or job)).tw,id. |
| 26 | (physician* or doctor* or clinician* or nurs* or hospitalist* or radiologists* or nephrologist* or therapist* or "medical staff" or cardiologist* or oncologist* or nutritionist* or pharmacist* or ophthalmologist* or otolaryngologist* or surgeon* or urologist* or epidemiologist* or pathologist* or physiatrist* or geriatrician* or gerontologist* or dentist* or doula* or anesthesiologist* or dermatologist*).tw,id. |
| 27 | ((hospital* or clinical or medical or health*) adj3 (staff or personnel or employee* or worker*)).tw,id. |
| 28 | exp health personnel/ or exp counselors/ or exp social workers/ or exp therapists/ |
| 29 | or/20-25 [Mental health, burnout, depression, anxiety] |
| 30 | or/26-28 [Health personnel] |
| 31 | 19 and 29 and 30 |
| 32 | limit 31 to (english language and yr="2003 -Current") |

Accessibility Information and TipsRevised Date: 07/2015

**Print Search History**

|  | Sunday, June 21, 2020 4:16:00 PM |
| --- | --- |

| **#** | **Query** |
| --- | --- |
| S53 | S32 AND S48 AND S50 |
| S52 | S32 AND S48 AND S50 |
| S51 | S32 AND S48 AND S50 |
| S50 | S33 OR S34 OR S35 OR S36 OR S37 OR S38 OR S39 OR S40 |
| S49 | S32 AND S44 AND S48 |
| S48 | S45 OR S46 OR S47 |
| S47 | TI ( (hospital* or clinical or medical or health*) N3 (staff or personnel or employee* or worker* or professional*) ) OR AB ( (hospital* or clinical or medical or health*) N3 (staff or personnel or employee* or worker* or professional*) ) |
| S46 | TI ( psychologist* or counsellor* or physician* or doctor* or clinician* or nurs* or hospitalist* or radiologists* or nephrologist* or therapist* or "medical staff" or cardiologist* or oncologist* or nutritionist* or pharmacist* or ophthalmologist* or otolaryngologist* or surgeon* or urologist* or epidemiologist* or pathologist* or physiatrist* or geriatrician* or gerontologist* or dentist* or doula* or anesthesiologist* or dermatologist* ) OR AB ( psychologist* or counsellor* or physician* or doctor* or clinician* or nurs* or hospitalist* or radiologists* or nephrologist* or therapist* or "medical staff" or cardiologist* or oncologist* or nutritionist* or pharmacist* or ophthalmologist* or otolaryngologist* or surgeon* or urologist* or epidemiologist* or pathologist* or physiatrist* or geriatrician* or gerontologist* or dentist* or doula* or anesthesiologist* or dermatologist* ) |
| S45 | (MH "Health Personnel+") |
| S44 | S41 OR S43 |
| S43 | S39 AND S42 |
| S42 | S36 OR S37 OR S38 OR S40 |
| S41 | S33 OR S34 OR S35 |
| S40 | (MH "Behavioral and Mental Disorders") OR (MH "Sleep Disorders+") |
| S39 | TI ( occupation* or work or profession* or job ) OR AB ( occupation* or work or profession* or job ) |
| S38 | TI ( behavio?r* or stress or adaptation or psychology or psychological or emotion* or depress* or attitude* or sleep or burnout or fatigue or compassion or resilience or mental* ) OR AB ( behavio?r* or stress or adaptation or psychology or psychological or emotion* or depress* or attitude* or sleep or burnout or fatigue or compassion or resilience or mental* ) |
| S37 | (MH "Mental Disorders+") |
| S36 | (MH "Stress, Psychological+") |
| S35 | (MH "Quality of Working Life") |
| S34 | (MH "Stress, Occupational") OR (MH "Compassion Fatigue") |
| S33 | TI ( (stress* or anxiety or anxious or burnout or burn-out or depress* or overwhelm* or "psychological workload" or fatigue* or exhaust*) N4 (occupation* or work or profession* or job) ) OR AB ( (stress* or anxiety or anxious or burnout or burn-out or depress* or overwhelm* or "psychological workload" or fatigue* or exhaust*) N4 (occupation* or work or profession* or job) ) |
| S32 | S5 OR S10 OR S11 OR S12 OR S17 OR S23 OR S24 OR S25 OR S26 OR S27 OR S28 OR S29 OR S30 OR S31 |
| S31 | TI coronavir* OR AB coronavir* |
| S30 | TI ( "Novel Corona virus" or "corona virus disease 2019" or "nCov 2019" or "SARS Coronavirus 2" or "COVID-2019" or nCoV or "2019-nCoV" or covid19 or " covid-19" or "SARS-CoV-2" ) OR AB ( "Novel Corona virus" or "corona virus disease 2019" or "nCov 2019" or "SARS Coronavirus 2" or "COVID-2019" or nCoV or "2019-nCoV" or covid19 or " covid-19" or "SARS-CoV-2" ) |
| S29 | TI ( "2020" N2 (pandemic or epidemic or "global disease spread" or "virus spread") ) OR AB ( "2020" N2 (pandemic or epidemic or "global disease spread" or "virus spread") ) |
| S28 | TX ( ((pandemic or epidemic or "global spread") N3 (coronavir* or corona or covid or "2020")) ) OR AB ( ((pandemic or epidemic or "global spread") N3 (coronavir* or corona or covid or "2020")) ) |
| S27 | TI ( ((new or novel or emerging or "2020") N2 (coronoviru* or "corona viru*")) ) OR AB ( ((new or novel or emerging or "2020") N2 (coronoviru* or "corona viru*")) ) |
| S26 | TI "Severe Acute Respiratory Syndrome Coronavirus 2" OR AB "Severe Acute Respiratory Syndrome Coronavirus 2" |
| S25 | TI ( "new coronaviru*" or "2019 ncov" or "nCov 2019" or "SARS Coronavirus 2" or "novel coronaviru*" ) OR AB ( "new coronaviru*" or "2019 ncov" or "nCov 2019" or "SARS Coronavirus 2" or "novel coronaviru*" ) |
| S24 | TI ( "corona viru*" or "nCov" or "2019 ncov" or "Novel Coronavirus" or "covid-19" or "SARS-COV-2" or "Severe Acute Respiratory Syndrome Coronavirus 2" or "coronavirus disease 2019" or "corona virus disease 2019" ) OR AB ( "corona viru*" or "nCov" or "2019 ncov" or "Novel Coronavirus" or "covid-19" or "SARS-COV-2" or "Severe Acute Respiratory Syndrome Coronavirus 2" or "coronavirus disease 2019" or "corona virus disease 2019" ) |
| S23 | S16 AND S22 |
| S22 | S18 OR S19 OR S20 OR S21 |
| S21 | (MH "Canada+") OR (MH "Mexico") OR (MH "United States+") |
| S20 | (MH "Europe+") |
| S19 | (MH "Korea") OR (MH "South Korea") |
| S18 | (MH "China+") |
| S17 | S13 AND S16 |
| S16 | S14 OR S15 |
| S15 | (MH "Coronavirus Infections") |
| S14 | (MH "Pneumonia+") |
| S13 | TX wuhan or beijing or shanghai or korea or italy or spain or usa or uk or brazil or france or mexico |
| S12 | TI ( ((wuhan or beijing or shanghai or korea or spain or portugal or italy or usa or uk or brazil or france or mexico) N2 corona*) ) OR AB ( ((wuhan or beijing or shanghai or korea or spain or portugal or italy or usa or uk or brazil or france or mexico) N2 corona*) ) |
| S11 | TI ( (2019-ncov or ncov19 or ncov-19 or 2019-novel CoV or sars-cov2 or sars-cov-2 or sarscov2 or sarscov-2 or Sars-coronavirus2 or Sars-coronavirus-2 or SARS-like coronavirus* or coronavirus-19 or covid19 or covid-19 or covid 2019 or ((novel or new or nouveau) N2 (CoV on nCoV or covid or coronavirus* or corona virus or Pandemi*2)) or ((covid or covid19 or covid-19) and pandemic*2) or (coronavirus* and pneumonia)) ) OR AB ( (2019-ncov or ncov19 or ncov-19 or 2019-novel CoV or sars-cov2 or sars-cov-2 or sarscov2 or sarscov-2 or Sars-coronavirus2 or Sars-coronavirus-2 or SARS-like coronavirus* or coronavirus-19 or covid19 or covid-19 or covid 2019 or ((novel or new or nouveau) N2 (CoV on nCoV or covid or coronavirus* or corona virus or Pandemi*2)) or ((covid or covid19 or covid-19) and pandemic*2) or (coronavirus* and pneumonia)) ) |
| S10 | S8 AND S9 |
| S9 | S6 OR S7 |
| S8 | TX wuhan |
| S7 | (MH "Pneumonia+") |
| S6 | TI ( pneumonia or covid* or coronavirus* or corona virus* or ncov* or 2019-ncov or sars* ) OR AB ( pneumonia or covid* or coronavirus* or corona virus* or ncov* or 2019-ncov or sars* ) |
| S5 | S3 NOT S4 |
| S4 | TX ( SARS or SARS-CoV or MERS or MERS-CoV or Middle East respiratory syndrome or camel* or dromedar* or equine or coronary or coronal or covidence* or covidien or influenza virus or HIV or bovine or calves or TGEV or feline or porcine or BCoV or PED or PEDV or PDCoV or FIPV or FCoV or SADS-CoV or canine or CCov or zoonotic or avian influenza or H1N1 or H5N1 or H5N6 or IBV or murine corona* ) OR TX ( SARS or SARS-CoV or MERS or MERS-CoV or Middle East respiratory syndrome or camel* or dromedar* or equine or coronary or coronal or covidence* or covidien or influenza virus or HIV or bovine or calves or TGEV or feline or porcine or BCoV or PED or PEDV or PDCoV or FIPV or FCoV or SADS-CoV or canine or CCov or zoonotic or avian influenza or H1N1 or H5N1 or H5N6 or IBV or murine corona* ) |
| S3 | S1 OR S2 |
| S2 | TI ( (coronavirus* or corona virus* or OC43 or NL63 or 229E or HKU1 or HCoV* or ncov* or covid* or sars-cov* or sarscov* or Sars-coronavirus* or Severe Acute Respiratory Syndrome Coronavirus*) ) OR AB ( (coronavirus* or corona virus* or OC43 or NL63 or 229E or HKU1 or HCoV* or ncov* or covid* or sars-cov* or sarscov* or Sars-coronavirus* or Severe Acute Respiratory Syndrome Coronavirus*) ) |
| S1 | (MH "Coronaviridae") OR (MH "Coronavirus") |

PROQUEST ERIC

Search Strategy

Set#: S1

Searched for: noft(coronavirus* or corona virus* or OC43 or NL63 or 229E or HKU1 or HCoV* or ncov* or covid* or sars-cov* or sarscov* or Sars-coronavirus* or Severe Acute Respiratory Syndrome Coronavirus*)

Databases: ERIC

Results: 2

Set#: S2

Searched for: noft(((wuhan or beijing or shanghai or korea or spain or portugal or italy or usa or uk or brazil or france or mexico) NEAR/2 corona*))

Databases: ERIC

Results: 0

Set#: S3

Searched for: noft(((wuhan OR beijing OR shanghai OR korea OR spain OR portugal OR italy OR usa OR uk OR brazil OR france OR mexico) NEAR/3 corona*))

Databases: ERIC

Results: 0

Set#: S4

Searched for: noft(((wuhan OR beijing OR shanghai OR korea OR spain OR portugal OR italy OR usa OR uk OR brazil OR france OR mexico) NEAR/4 corona*))

Databases: ERIC

Results: 0

Set#: S5

Searched for: noft(((wuhan or beijing or shanghai or korea or spain or portugal or italy or USA or UK or brazil or france or mexico) NEAR/2 pneumoni*).)

Databases: ERIC

Results: 0

Set#: S6

Searched for: noft("corona viru*" or "nCov" or "2019 ncov" or "Novel Coronavirus" or "covid-19" or "SARS-COV-2" or "Severe Acute Respiratory Syndrome Coronavirus 2" or "coronavirus disease 2019" or "corona virus disease 2019")

Databases: ERIC

Results: 0

Set#: S7

Searched for: noft("new coronaviru*" or "2019 ncov" or "nCov 2019" or "SARS Coronavirus 2" or "novel coronaviru*")

Databases: ERIC

Results: 0

Set#: S8

Searched for: noft(((new or novel or emerging or "2020") NEAR/2 (coronoviru* or "corona viru*")))

Databases: ERIC

Results: 0

Set#: S9

Searched for: noft((pandemic or epidemic or "global spread") NEAR/3 (coronavir* or corona or covid or "2020"))

Databases: ERIC

Results: 0

Set#: S10

Searched for: noft("2020" NEAR/2 (pandemic or epidemic or "global disease spread" or "virus spread"))

Databases: ERIC

Results: 0

Set#: S11

Searched for: noft("Novel Corona virus" or "corona virus disease 2019" or "nCov 2019" or "SARS Coronavirus 2" or "COVID-2019" or nCoV or "2019-nCoV" or covid19 or " covid-19" or "SARS-CoV-2")

Databases: ERIC

Results: 0

Set#: S12

Searched for: noft(coronavir*)

Databases: ERIC

Results: 1

Set#: S13

Searched for: noft(covid)

Databases: ERIC

Results: 0

Set#: S14

Searched for: noft((stress* or anxiety or anxious or burnout or burn-out or depress* or overwhelm* or "psychological workload" or fatigue* or exhaust*) NEAR/4 (occupation* or work or profession* or job))

Databases: ERIC

Results: 3702

Set#: S15

Searched for: noft((behavio?r* or stress* or adaptation or psycholog* or psychological or emotion* or depress* or attitude* or sleep or burnout or fatigue* or compassion or resilience or resilient* or anxiety or anxious* or exhaust* or mental).)

Databases: ERIC

Results: 637978

Set#: S16

Searched for: noft(physician* or counsellor* or psychologist* or doctor* or clinician* or nurs* or hospitalist* or radiologists* or nephrologist* or therapist* or "medical staff" or cardiologist* or oncologist* or nutritionist* or pharmacist* or ophthalmologist* or otolaryngologist* or surgeon* or urologist* or epidemiologist* or pathologist* or physiatrist* or geriatrician* or gerontologist* or dentist* or doula* or anesthesiologist* or dermatologist*)

Databases: ERIC

Results: 73052

Set#: S17

Searched for: noft(((hospital* or clinical or medical or health*) NEAR/3 (staff or personnel or employee* or worker* or professional*)).)

Databases: ERIC

Results: 13985

Set#: S18

Searched for: noft(SARS or SARS-CoV or MERS or MERS-CoV or Middle East respiratory syndrome or camel* or dromedar* or equine or coronary or coronal or covidence* or covidien or influenza virus or HIV or bovine or calves or TGEV or feline or porcine or BCoV or PED or PEDV or PDCoV or FIPV or FCoV or SADS-CoV or canine or CCov or zoonotic or avian influenza or H1N1 or H5N1 or H5N6 or IBV or murine corona*)

Databases: ERIC

Results: 4080

Set#: S19

Searched for: noft(coronavirus* OR corona virus* OR OC43 OR NL63 OR 229E OR HKU1 OR HCoV* OR ncov* OR covid* OR sars-cov* OR sarscov* OR Sars-coronavirus* OR Severe Acute Respiratory Syndrome Coronavirus*) OR noft(((wuhan OR beijing OR shanghai OR korea OR spain OR portugal OR italy OR usa OR uk OR brazil OR france OR mexico) NEAR/2 corona*)) OR noft(((wuhan OR beijing OR shanghai OR korea OR spain OR portugal OR italy OR usa OR uk OR brazil OR france OR mexico) NEAR/3 corona*)) OR noft(((wuhan OR beijing OR shanghai OR korea OR spain OR portugal OR italy OR usa OR uk OR brazil OR france OR mexico) NEAR/4 corona*)) OR noft(((wuhan OR beijing OR shanghai OR korea OR spain OR portugal OR italy OR USA OR UK OR brazil OR france OR mexico) NEAR/2 pneumoni*) .) OR noft("corona viru*" OR "nCov" OR "2019 ncov" OR "Novel Coronavirus" OR "covid-19" OR "SARS-COV-2" OR "Severe Acute Respiratory Syndrome Coronavirus 2" OR "coronavirus disease 2019" OR "corona virus disease 2019") OR noft("new coronaviru*" OR "2019 ncov" OR "nCov 2019" OR "SARS Coronavirus 2" OR "novel coronaviru*") OR noft(((new OR novel OR emerging OR "2020") NEAR/2 (coronoviru* OR "corona viru*"))) OR noft((pandemic OR epidemic OR "global spread") NEAR/3 (coronavir* OR corona OR covid OR "2020")) OR noft("2020" NEAR/2 (pandemic OR epidemic OR "global disease spread" OR "virus spread")) OR noft("Novel Corona virus" OR "corona virus disease 2019" OR "nCov 2019" OR "SARS Coronavirus 2" OR "COVID-2019" OR nCoV OR "2019-nCoV" OR covid19 OR " covid-19" OR "SARS-CoV-2") OR noft(coronavir*) OR noft(covid)

Databases: ERIC

These databases are searched for part of your query.

Results: 2

Set#: S20

Searched for: (noft(coronavirus* OR corona virus* OR OC43 OR NL63 OR 229E OR HKU1 OR HCoV* OR ncov* OR covid* OR sars-cov* OR sarscov* OR Sars-coronavirus* OR Severe Acute Respiratory Syndrome Coronavirus*) OR noft(((wuhan OR beijing OR shanghai OR korea OR spain OR portugal OR italy OR usa OR uk OR brazil OR france OR mexico) NEAR/2 corona*)) OR noft(((wuhan OR beijing OR shanghai OR korea OR spain OR portugal OR italy OR usa OR uk OR brazil OR france OR mexico) NEAR/3 corona*)) OR noft(((wuhan OR beijing OR shanghai OR korea OR spain OR portugal OR italy OR usa OR uk OR brazil OR france OR mexico) NEAR/4 corona*)) OR noft(((wuhan OR beijing OR shanghai OR korea OR spain OR portugal OR italy OR USA OR UK OR brazil OR france OR mexico) NEAR/2 pneumoni*) .) OR noft("corona viru*" OR "nCov" OR "2019 ncov" OR "Novel Coronavirus" OR "covid-19" OR "SARS-COV-2" OR "Severe Acute Respiratory Syndrome Coronavirus 2" OR "coronavirus disease 2019" OR "corona virus disease 2019") OR noft("new coronaviru*" OR "2019 ncov" OR "nCov 2019" OR "SARS Coronavirus 2" OR "novel coronaviru*") OR noft(((new OR novel OR emerging OR "2020") NEAR/2 (coronoviru* OR "corona viru*"))) OR noft((pandemic OR epidemic OR "global spread") NEAR/3 (coronavir* OR corona OR covid OR "2020")) OR noft("2020" NEAR/2 (pandemic OR epidemic OR "global disease spread" OR "virus spread")) OR noft("Novel Corona virus" OR "corona virus disease 2019" OR "nCov 2019" OR "SARS Coronavirus 2" OR "COVID-2019" OR nCoV OR "2019-nCoV" OR covid19 OR " covid-19" OR "SARS-CoV-2") OR noft(coronavir*) OR noft(covid)) NOT noft(SARS OR SARS-CoV OR MERS OR MERS-CoV OR Middle East respiratory syndrome OR camel* OR dromedar* OR equine OR coronary OR coronal OR covidence* OR covidien OR influenza virus OR HIV OR bovine OR calves OR TGEV OR feline OR porcine OR BCoV OR PED OR PEDV OR PDCoV OR FIPV OR FCoV OR SADS-CoV OR canine OR CCov OR zoonotic OR avian influenza OR H1N1 OR H5N1 OR H5N6 OR IBV OR murine corona*)

Databases: ERIC

These databases are searched for part of your query.

Results: 0

Set#: S21

Searched for: noft((stress* OR anxiety OR anxious OR burnout OR burn-out OR depress* OR overwhelm* OR "psychological workload" OR fatigue* OR exhaust*) NEAR/4 (occupation* OR work OR profession* OR job)) OR noft((behavio?r* OR stress* OR adaptation OR psycholog* OR psychological OR emotion* OR depress* OR attitude* OR sleep OR burnout OR fatigue* OR compassion OR resilience OR resilient* OR anxiety OR anxious* OR exhaust* OR mental) .)

Databases: ERIC

These databases are searched for part of your query.

Results: 638057

Set#: S22

Searched for: noft(physician* OR counsellor* OR psychologist* OR doctor* OR clinician* OR nurs* OR hospitalist* OR radiologists* OR nephrologist* OR therapist* OR "medical staff" OR cardiologist* OR oncologist* OR nutritionist* OR pharmacist* OR ophthalmologist* OR otolaryngologist* OR surgeon* OR urologist* OR epidemiologist* OR pathologist* OR physiatrist* OR geriatrician* OR gerontologist* OR dentist* OR doula* OR anesthesiologist* OR dermatologist*) OR noft(((hospital* OR clinical OR medical OR health*) NEAR/3 (staff OR personnel OR employee* OR worker* OR professional*)) .)

Databases: ERIC

These databases are searched for part of your query.

Results: 81831

Set#: S23

Searched for: (noft(coronavirus* OR corona virus* OR OC43 OR NL63 OR 229E OR HKU1 OR HCoV* OR ncov* OR covid* OR sars-cov* OR sarscov* OR Sars-coronavirus* OR Severe Acute Respiratory Syndrome Coronavirus*) OR noft(((wuhan OR beijing OR shanghai OR korea OR spain OR portugal OR italy OR usa OR uk OR brazil OR france OR mexico) NEAR/2 corona*)) OR noft(((wuhan OR beijing OR shanghai OR korea OR spain OR portugal OR italy OR usa OR uk OR brazil OR france OR mexico) NEAR/3 corona*)) OR noft(((wuhan OR beijing OR shanghai OR korea OR spain OR portugal OR italy OR usa OR uk OR brazil OR france OR mexico) NEAR/4 corona*)) OR noft(((wuhan OR beijing OR shanghai OR korea OR spain OR portugal OR italy OR USA OR UK OR brazil OR france OR mexico) NEAR/2 pneumoni*) .) OR noft("corona viru*" OR "nCov" OR "2019 ncov" OR "Novel Coronavirus" OR "covid-19" OR "SARS-COV-2" OR "Severe Acute Respiratory Syndrome Coronavirus 2" OR "coronavirus disease 2019" OR "corona virus disease 2019") OR noft("new coronaviru*" OR "2019 ncov" OR "nCov 2019" OR "SARS Coronavirus 2" OR "novel coronaviru*") OR noft(((new OR novel OR emerging OR "2020") NEAR/2 (coronoviru* OR "corona viru*"))) OR noft((pandemic OR epidemic OR "global spread") NEAR/3 (coronavir* OR corona OR covid OR "2020")) OR noft("2020" NEAR/2 (pandemic OR epidemic OR "global disease spread" OR "virus spread")) OR noft("Novel Corona virus" OR "corona virus disease 2019" OR "nCov 2019" OR "SARS Coronavirus 2" OR "COVID-2019" OR nCoV OR "2019-nCoV" OR covid19 OR " covid-19" OR "SARS-CoV-2") OR noft(coronavir*) OR noft(covid)) AND (noft((stress* OR anxiety OR anxious OR burnout OR burn-out OR depress* OR overwhelm* OR "psychological workload" OR fatigue* OR exhaust*) NEAR/4 (occupation* OR work OR profession* OR job)) OR noft((behavio?r* OR stress* OR adaptation OR psycholog* OR psychological OR emotion* OR depress* OR attitude* OR sleep OR burnout OR fatigue* OR compassion OR resilience OR resilient* OR anxiety OR anxious* OR exhaust* OR mental) .)) AND (noft(physician* OR counsellor* OR psychologist* OR doctor* OR clinician* OR nurs* OR hospitalist* OR radiologists* OR nephrologist* OR therapist* OR "medical staff" OR cardiologist* OR oncologist* OR nutritionist* OR pharmacist* OR ophthalmologist* OR otolaryngologist* OR surgeon* OR urologist* OR epidemiologist* OR pathologist* OR physiatrist* OR geriatrician* OR gerontologist* OR dentist* OR doula* OR anesthesiologist* OR dermatologist*) OR noft(((hospital* OR clinical OR medical OR health*) NEAR/3 (staff OR personnel OR employee* OR worker* OR professional*)) .))

Databases: ERIC

These databases are searched for part of your query.

Results: 0

----------------------------------------------------------
